# Co-expression of PADI isoforms during progenitor differentiation enables functional diversity

**DOI:** 10.1098/rstb.2022.0451

**Published:** 2023-11-20

**Authors:** Kim Vikhe Patil, Mandy Meijer, Kylie Hin-Man Mak, Wei Yang, Ana Mendanha Falcão, Gonçalo Castelo-Branco, Maria Genander

**Affiliations:** ^1^ Department of Cell and Molecular Biology, Karolinska Institutet, Stockholm, Sweden; ^2^ Laboratory of Molecular Neurobiology, Department of Medical Biochemistry and Biophysics, Karolinska Institutet, Stockholm, Sweden; ^3^ Ming Wai Lau Center for Reparative Medicine, Stockholm Node, Karolinska Institutet, Stockholm, Sweden; ^4^ Life and Health Sciences Research Institute (ICVS), School of Medicine, University of Minho, Braga, Portugal; ^5^ ICVS/3B's Associate Laboratory, PT Government Associate Laboratory, 4710-057 Braga/Guimarães, Portugal

**Keywords:** isoform, *Padi* *2*, *Padi* *3*, hair follicle, oligodendrocyte, citrullination

## Abstract

Protein isoforms, generated through alternative splicing or promoter usage, contribute to tissue function. Here, we characterize the expression of predicted *Padi3α* and *Padi3β* isoforms in hair follicles and describe expression of *Padi2β*, a hitherto unknown PADI2 isoform, in the oligodendrocyte lineage. *Padi2β* transcription is initiated from a downstream intronic promoter, generating an N-terminally truncated, unstable, PADI2β. By contrast to the established role of the canonical PADI2 (PADI2α) (Falcao *et al*. 2019 *Cell Rep*. **27**, 1090–1102.e10. (doi:10.1016/j.celrep.2019.03.108)), PADI2β inhibits oligodendrocyte differentiation, suggesting that PADI2 isoforms exert opposing effects on oligodendrocyte lineage progression. We localize *Padi3α* and *Padi3β* to developing hair follicles and find that both transcripts are expressed at low levels in progenitor cells, only to increase in expression concomitant with differentiation. When expressed *in vitro*, PADI3α and PADI3β are enriched in the cytoplasm and precipitate together. Whereas PADI3β protein stability is low and PADI3β fails to induce protein citrullination, we find that the enzymatic activity and protein stability of PADI3α is reduced in the presence of PADI3β. We propose that PADI3β modulates PADI3α activity by direct binding and heterodimer formation. Here, we establish expression and function of *Padi2* and *Padi3* isoforms, expanding on the mechanisms in place to regulate citrullination in complex tissues.

This article is part of the Theo Murphy meeting issue ‘The virtues and vices of protein citrullination’.

## 1. Introduction

Development and maintenance of tissues require temporal as well as cell type-specific coordination of gene expression. In addition, alternative splicing and differential promoter usage contribute to diversify mRNA species, or isoforms, generated from single genes, which eventually affect protein structure. As a result, protein isoforms often exert distinct or even opposing functions. Transcriptional effector isoforms transcribed from the same gene are demonstrated to display distinct DNA binding specificity and cellular location [1–4]. Alternative splicing of transmembrane receptors impact on ligand binding affinity [5] and isoforms of post-translational modifiers, including kinases and acetylases, have distinct substrate specificity [6,7]. Global changes in transcript isoform signatures are described both in stem cell lineage progression and cancer [8,9]. In addition, splicing is deregulated in tumor cells [10] and alternative promoter usage is predictive of cancer patient survival [11] reflecting the broad functional impact of protein isoforms on both tissue development and disease.

Peptidylarginine deiminases (PADIs) are associated with a growing number of processes including stem cell maintenance and lineage progression [12–15], inflammatory diseases [16–19] and cancer [20]. PADIs mediate Ca^2+^-dependent citrullination; a post-translational enzymatic conversion of the positively charged amino acid arginine to the neutral citrulline. Citrullination leads to a decreased net protein charge affecting folding, binding affinity and in case of histone-tail citrullination, chromatin accessibility [21]. There are currently five mammalian PADIs described which despite having a high sequence homology, differ in their subcellular localization, dimerization capacity and substrate specificity [22–24]. Moreover, additional mouse and human PADI isoforms, generated through alternative splicing and/or promoter usage are predicted [25] but hitherto never validated. In mouse, the *Padi3* orthologue is annotated to produce two protein-coding transcripts, whereas both *PADI2* and *PADI4* are predicted to generate dual isoforms in humans. These predictions are intriguing in suggesting that PADI isoform diversity is relatively common and could act to modulate the functional outcome or activity of the PADI family in multiple cellular contexts.

**Figure 1.**
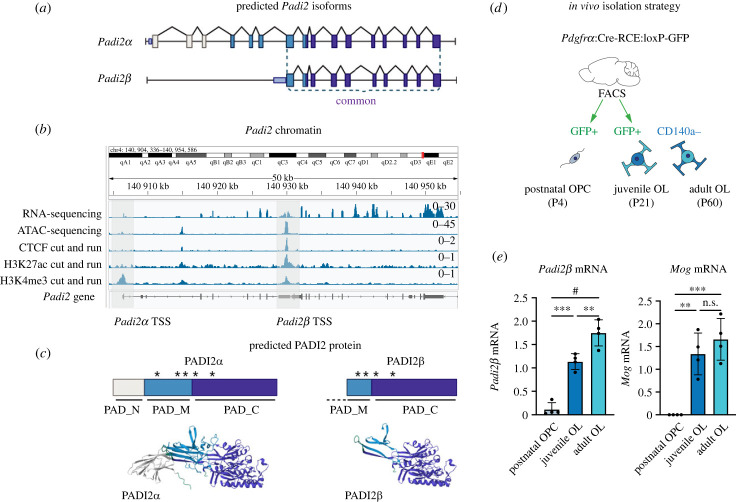
Identification and characterization of *Padi2* isoform expression. (*a*) Schematic illustration of *Padi2α* and *Padi2β* transcripts identified by RNA sequencing and PCR walking, highlighting exons (boxes) and introns (lines). *Padi2β* transcription is initiated downstream within an intronic region, generating a significantly truncated mRNA and protein. (*b*) Sequencing and epigenetic profiling of *Padi2* chromatin in primary oligodendrocytes [26] uncovers an accessible intronic promoter region (bound by CTCF and enriched for H3K27ac and H3K4me3) upstream of exon 7 in the *Padi2* gene, corresponding to the *Padi2β* transcription start site (TSS). (*c*) Simplified diagram of the PADI2 protein structures to illustrate the lack of the PAD_N and parts of PAD_M domains in PADI2β (right), with three-dimensional models underneath. Representative protein structures illustrating the differences between the two PADI2 protein isoforms are shown based on AlphaFold's PADI2α prediction. (*d*) Schematic of FACS strategy for oligodendrocyte populations. The *Pdgfrα*-Cre-RCE:loxP-GFP-loxP reporter mouse labels oligodendrocyte precursors (P4) (GFP+) as well as juvenile (P21) and adult (P60) GFP + but CD140a-negative oligodendrocytes. (*e*) mRNA expression of *Padi2β* in the oligodendrocyte populations described in (h). *Padi2β* levels increase with oligodendrocyte differentiation, corresponding to increased expression of the oligodendrocyte differentiation marker *Mog,* (*n* = 4). Data are represented as mean ± s.d.; **p < 0.01, ***p < 0.001, #*p* < 0.0001 using multiple unpaired *t*-test.

PADIs share a common protein structure and are composed of three subdomains, two N-terminal IgG domains (PAD_N and PAD_M) and a C-terminal composed of a conserved *α*/*β* propeller domain containing the active site cleft (PAD_C) (figures 1*c* and 3*b*) [27]. Ca^2+^ binding sites required for enzymatic activity are located near the active cleft in the C-terminal and middle IgG domain, and Ca^2+^ binding is believed to induce conformational change mediating recognition of the substrate [27]. The most N-terminal IgG domain is likely to be involved in protein binding—both in PADI homodimer formation as well as interaction with other proteins [27,28].

Despite the relatively well-characterized function of each PAD domain, spatially dispersed mutations affecting the structure of different domains have a similar impact on PADI properties. As such, human PADI3 point mutations targeting three conserved amino acid residues (L112, A294 and P605) are associated with hair shaft differentiation defects [29]. *In vitro* characterization indicates that PADI3 protein stability as well as enzymatic activity are reduced upon mutation of either residue [29], suggesting that similar aspects of PADI3 functionality are affected irrespective of the location of the mutation within the protein.

Given the unexplored nature of PADI isoforms in general and the functional importance of small structural changes in PADIs in particular [29], we probed the expression and function of *Padi2* and *Padi3* isoforms using two progenitor lineages where the roles of PADI2/3 are well established [12,14]. Using *in vivo* isolated oligodendrocyte lineage cells, we identify expression of an uncharacterized *Padi2* transcript, *Padi2β,* and find that the levels of *Padi2β* increase during oligodendrocyte lineage maturation and differentiation. Although PADI2β protein abundance is low in cultured oligodendrocyte precursors, silencing of *Padi2β* promotes oligodendrocyte differentiation, indicating that the canonical PADI2 isoform (PADI2α) and PADI2β have opposing functions in oligodendrocyte lineage progression [12]. Furthermore, we identify and localize both the canonical (*Padi3α*) and the predicted *Padi3* (*Padi3β*) transcript isoforms to hair follicle progenitor as well as lineage-committed cells *in vivo.* While PADI3β protein is unstable and fails to induce citrullination, PADI3β can bind PADI3α, thereby negatively impacting the enzymatic activity of PADI3α.

Exploiting the oligodendrocyte, as well as hair follicle, progenitor lineage [12,14], we demonstrate not only that *Padi* isoforms are expressed *in vivo* but unveil how the relative isoform abundancy could modify the functional outcome of PADI enzyme activity in developing and adult tissues.

## 2. Results

### (a) *Padi2* isoforms are expressed in the oligodendrocyte lineage

Previous work identified PADI2 as an epigenetic mediator of oligodendrocyte precursor differentiation as well as a facilitator of oligodendrocyte myelination [12]. Reanalysis of available RNA-sequencing data from primary oligodendrocyte precursors suggested, and PCR walking confirmed, expression of an additional, truncated, *Padi2* mRNA (*Padi2β*) (figure 1*a,b*) (see reference [30] for original RNA-sequencing data set). *Padi2β* transcription is initiated from a downstream accessible intronic region bound by the transcriptional activator and genome architectural protein CTCF (CCCTC-binding factor) and enriched for H3K27ac and H3K4me3 marks [30], collectively indicating transcriptional activity from an alternative promoter (figure 1*b*). The downstream intronic *Padi2β* transcriptional start site results in a shorter, N-terminally truncated, 449 amino acids long PADI2β isoform (compared to the 673 amino acids long PADI2α) (figure 1*c*). In addition, the two initial PADI2β amino acids (M and D) are isoform specific, replacing the first two codons in *Padi2α* exon 7 (corresponding to amino acids E and N) (electronic supplementary material, figure S1a). The resulting PADI2β protein is stipulated to lack the entire N-terminal (PAD_N) and parts of the middle (PAD_M) domain including a Ca^2+^ binding site. As no predicted three-dimensional model of PADI2β is available [1,12], the polypeptide structure corresponding to the truncated genomic sequence was extracted from the three-dimensional structure of PADI2α to provide a visual representation of the truncated PADI2β protein (figure 1*c*).

Taking advantage of a *Pdgfrα*-Cre-RCE:loxP-GFP-loxP reporter mouse [12], where GFP expression marks progeny derived from developing *Pdgfrα*-positive oligodendrocyte precursors, we FACS purified postnatal oligodendrocyte precursors (P4) as well as juvenile (P21) and mature (P60) oligodendrocytes (figure 1*d*). In line with previous reports characterizing *Padi2α* expression [12], we found induction of *Padi2β* and *Mog* (a marker for oligodendrocyte differentiation) mRNA during oligodendrocyte lineage maturation, indicating that *Padi2β* transcripts are dynamically regulated *in vivo* during oligodendrocyte lineage progression mimicking the expression dynamics reported for *Padi2α* (as shown in [12]) (figure 1*e*). Our data indicate that *Padi2α* and *Padi2β* are co-regulated in the oligodendrocyte lineage and that *Padi2β* transcription is initiated through a downstream intragenic alternative promoter region.

### (b) PADI2β is unstable, yet inhibits oligodendrocyte differentiation

**Figure 2.**
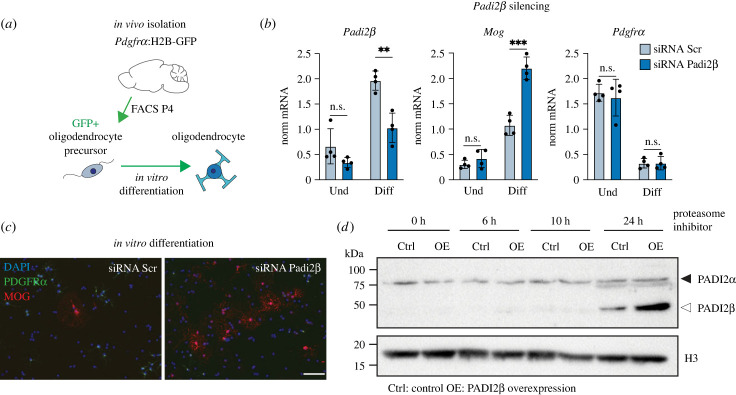
PADI2β inhibits oligodendrocyte differentiation. (*a*) *Pdgfrα*:H2B-GFP positive oligodendrocyte precursors were FACS isolated at P4 and subsequently differentiated for 2.5 days *in vitro* into mature oligodendrocytes. (*b*) Silencing of *Padi2β* is efficient in differentiated oligodendrocytes (as shown in left graph). *Mog* mRNA levels (middle graph) are increased in differentiating siPadi2β target cells, when compared to scrambled control siRNA (Scr) targeted precursors. *Pdgfrα* (a precursor marker) is downregulated in both control and siPadi2β targeted oligodendrocytes (right). Data are represented as mean ± s.d.; ns not significant, **p < 0.01, ***p < 0.001 using multiple unpaired *t*-test. (*c*) Immunofluorescent images of 2.5 days *in vitro* differentiated primary oligodendrocyte precursor cells reveal a higher occurrence of MOG-positive (differentiated) cells upon *Padi2β* silencing when compared to control siRNA targeted cells. Scale bar = 50 µm. (*d*) Treatment with proteasome inhibitor MG132 (10 µM) for 0–24 h of either PADI2β overexpressing (OE) or control (Ctrl) Oli-neu cells results in the accumulation of PADI2β (white arrow) in both cell types at 24 h. Histone H3 is used as internal loading control. Blot is representative of three independent experiments.

PADI2α promotes oligodendrocyte precursor differentiation [12]. To understand if PADI2β mirrors the function of PADI2α, we cultured *in vivo* isolated, *Pdgfrα*:H2B-GFP positive, oligodendrocyte precursors (figure 2*a*) and probed their ability to differentiate *in vitro* upon silencing of *Padi2β*. Comparing control (Scrambled) siRNA and siPadi2β targeted oligodendrocyte precursors in differentiation conditions, we found that *Padi2β* silenced precursors were more prone to differentiate as shown by increased expression of *Mog* as well as a higher occurrence of MOG positive oligodendrocytes (figure 2*b*,*c*). To corroborate the contrasting roles of PADI2α and PADI2β on oligodendrocyte differentiation, we silenced *Padi2α* and *Padi2β* in parallel using the oligodendrocyte precursor cell line (Olineu) [31] and evaluated the effect on differentiation compared to precursors targeted with a control siRNA. These experiments confirmed that PADI2α and PADI2β have opposing effects on oligodendrocyte precursor differentiation, where PADI2β acts to restrict lineage progression (electronic supplementary material, figure S1*b*,*c*).

Probing for potential compensation between the two *Padi2* isoforms revealed that silencing of *Padi2α* reduced *Padi2β* levels, whereas siRNA targeting of *Padi2β* failed to affect *Padi2α* expression (electronic supplementary material, figure S1*b*,*c*). Exploiting an siRNA targeting the shared *Padi2* 3′UTR sequence, we found that silencing of both *Padi2α* and *Padi2β* promoted oligodendrocyte differentiation as judged by *Mog* expression (electronic supplementary material, figure S1*d*). Taken together, these data confirm that the *Padi2* isoforms play opposing roles in oligodendrocyte differentiation and reveal that combined silencing of *Padi2α* and *Padi2β* phenocopy differentiation dynamics of *Padi2β* siRNA targeted oligodendrocyte precursors.

Arguing that expression of PADI2β could inhibit oligodendrocyte differentiation by balancing the action of PADI2α, we generated a PADI2β-overexpressing oligodendrocyte precursor cell line [31]. Comparing PADI2α and PADI2β protein levels, using a previously published antibody able to faithfully capture both PADI2 overexpression and knock out [12,32–35], revealed enrichment of PADI2α but not PADI2β, neither in control nor in PADI2β-overexpressing cells (figure 2*d*, 0 h). Reasoning that PADI2β could be rapidly degraded, we added a proteasome inhibitor for 6, 10 or 24 h to both control and PADI2β-overexpressing oligodendrocyte precursors. Proteasomal inhibition resulted in the accumulation of the shorter PADI2β isoform in control as well as in PADI2-overexpressing precursors (figure 2*d*, 24 h). To verify the identity of the relatively weak PADI2α immunoreactivity seen on western blot, we repeated the experiment using differentiated oligodendrocytes, a setting where the baseline expression of *Padi2α* is increased (electronic supplementary material, figure S1*b*–*d*). We found that PADI2α was firmly expressed in differentiated oligodendrocytes, confirming the previously demonstrated [12,32–35] specificity of the PADI2 antibody (electronic supplementary material, figure S1*e*). These data suggest that the N-terminal truncation of PADI2β affects PADI2β stability and that transient, or low abundant, expression of PADI2β can act to modulate the effects of PADI2α in oligodendrocyte lineage differentiation.

### (c) *Padi3α* and *Padi3β* are co-expressed in developing hair follicles

**Figure 3.**
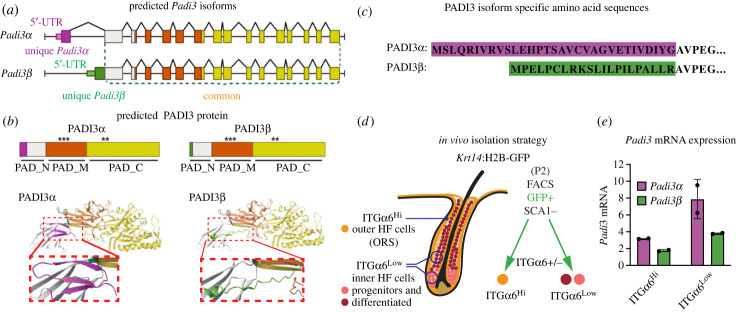
Characterization of *Padi3* isoform expression. (*a*) Schematic illustration of *Padi3α* and the predicted *Padi3β* chromatin highlighting exons (boxes) and introns (lines). *Padi3α* and *Padi3β* first exon with appended 5′UTRs are coloured in purple and green, respectively. *Padi3β* transcription is initiated downstream of the first exon of *Padi3α*, and the *Padi3β* mRNA begins with the intronic region (green box) being combined with a part of the second *Padi3α* exon (large white box). (*b*) PADI3α and PADI3β differ in their N-terminal domain (PAD_N), whereas the middle (PAD_M) and C-terminal (PAD_C) domain are identical. Calcium binding sites (*), required for enzymatic activity are intact in both PADI3 isoforms. Three-dimensional models (Q9Z184 for PADI3α and E9QAM4 for PADI3β) generated using AlphaFold prediction [36,37] show the difference in the N-terminal truncation in PADI3β (highlighted in green) compared with PADI3α (highlighted in purple). (*c*) The amino acid sequences of the unique portion of each PADI3 isoform, colour coding corresponds to the structure highlighted in (*a*,*b*). (*d*) Illustration of FACS strategy using a *Krt14*:H2B-GFP mouse at P2 to mark all epithelial cells (interfollicular epidermis and hair follicle). SCA1 expression is used to discern hair follicle epithelium (SCA1-negative) from interfollicular epidermis (SCA1-positive). Two hair follicle cell populations (GFP+, SCA1-) are FACS isolated based on ITGα6 (integrin-α6) expression. ITGα6-positive cells (ITGα6^Hi^) correspond to hair follicle stem cell progeny located at the basement membrane along the hair follicle (outer HF cells (ORS (outer root sheath)), whereas ITGα6-negative (ITGα6^Low^) cells reside in the inner cell layers of the hair follicle and represent progenitors and differentiated hair lineages (inner HF cells). (*e*) *Padi3α* and *Padi3β* mRNA levels comparing ITGα6^Low^ and ITGα6^Hi^ hair follicle cells, showing the normalized mRNA expression relative to *Padi3α* and *Padi3β* expression in primary keratinocytes in culture. Both *Padi3α* and *Padi3β* are enriched in the inner cell layers of the hair follicle (ITGα6^Low^), corresponding to progenitor and differentiated hair lineage cells, (*n* = 2). Data are represented as mean ± s.d.

Although the existence of mouse, but not human, *Padi3* isoforms are predicted, no experimental demonstration of expression and function is available. To this end, we characterized the two annotated *Padi3* transcripts, where *Padi3α* represents the canonical (ENSMUST00000026377.9), and *Padi3β* the uncharacterized isoform (ENSMUST00000172098.2) (figure 3*a*). *Padi3β* transcription is initiated downstream of the canonical *Padi3α* transcription start and the resulting *Padi3β* mRNA replaces the first *Padi3α* coding exon with part of the neighbouring intron (figure 3*a*). As a result, *Padi3α* and *Padi3β* generate proteins of 664 and 654 amino acids, respectively, differing only in the very beginning of the N-terminus (figure 3*b*,*c*). Three-dimensional structural prediction of the PADI3β protein (UniProt E9QAM4) suggests that the altered N-terminus in PADI3β is less complex when compared to PADI3α (UniProt Q9Z184), as it lacks an ordered secondary structure (figure 3*b*) [36,37].

To understand if *Padi3α* and *Padi3β* are expressed *in vivo*, we turned to the developing hair follicles, a tissue where PADI3 expression is prominent and well established [14,26,38]. Hair follicle progenitor and differentiated cells were FACS isolated at postnatal day 2 (P2), a time point when hair shaft differentiation is initiated and *Padi3* is highly expressed. Using the *Krt14*:H2B-GFP mouse line to select for epithelial skin cells, two hair follicle cell populations were FACS isolated based on integrin-α6 (ITGα6) expression. ITGα6-positive hair follicle cells correspond to an outer layer of hair follicle stem cell progeny (ORS, outer root sheath) whereas the ITGα6-negative cells are found in the inner cell layers of the hair follicle and correspond to proliferating progenitor cells and differentiated hair lineage cells (hair shaft and IRS (inner root sheath)) [39,40] (figure 3*d*). Designing primers specific for each predicted *Padi3* isoform, we performed RT-qPCR on RNA extracted from the sorted ITGα6-positive and ITGα6-negative cell populations. We detected *Padi3α* as well as *Padi3β* mRNA in both hair follicle cell populations, where expression of both *Padi3* isoforms was enriched in the cells derived from the inner layers, in line with the previously described function of PADI3 in hair shaft differentiation [29,41–44]. These data suggest that both *Padi3α* and *Padi3β* are expressed in the hair follicle progenitor cell lineage during development.

### (d) Localization and function of *Padi3* isoforms

**Figure 4.**
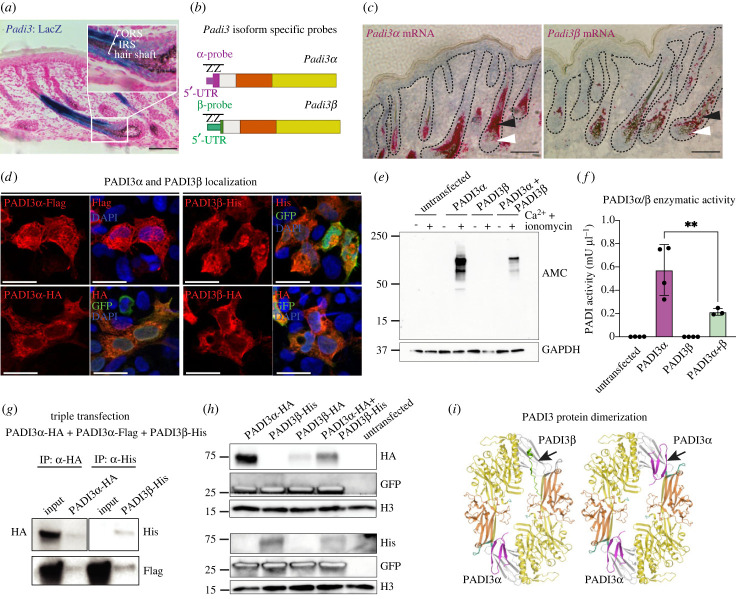
*In vivo Padi3* isoform co-expression and function. (*a*) X-gal stain of the Padi3^tm1a^ :LacZ reporter mouse show PADI3 protein expression localized predominantly to the differentiating cell populations of the IRS and hair shaft. Weak LacZ reactivity is also observed in progenitor cells in the lower part of the hair follicle. The ORS is largely LacZ-negative. ORS, outer root sheath; IRS, inner root sheath; scale bar = 100 µm. (*b*) Schematic illustration of the design strategy of BaseScope probes specific for either *Padi3* mRNA isoform. The different probes bind the unique parts of each mRNA (purple for *α*, green for *β*), including the 5′UTR. (*c*) BaseScope *in situ* hybridization display that both *Padi3α* and *Padi3β* are detected in the early neonatal (P2) hair follicle. Both isoforms are co-expressed in the differentiated cell populations of the hair follicle (black arrowheads) as well as in the progenitor population (white arrowheads), albeit at lower levels. Scale bar = 100 µm. (*d*) Immunofluorescent images of HEK293 48 h after transfection of tagged PADI3α and PADI3β. Both PADI3α and PADI3β localize to the cytoplasm (Flag and HA for PADI3α, and His and HA for PADI3β). The vector backbones (except for Flag) also code for GFP, which is used as a proxy for transfection efficiency. Scale bar = 25 µm. (*e*) Western blot analysis of modified citrulline (AMC, anti-modified citrulline) in HEK293 cells expressing either PADI3α or PADI3β alone, or PADI3α and PADI3β in combination. Cells were stimulated with 5 mM Ca^2+^ and 5 µM Ionomycin (+) or DMSO (−) for 3 h. Notably, co-expression of PADI3α or PADI3β results in reduced citrullination signal when compared to HEK293 cells transfected with PADI3α alone. GAPDH is used as internal loading control. Blot is representative of two independent experiments. (*f*) Analysis of the citrullinating ability of PADI3α and PADI3β alone or in combination, using the Antibody-Based Assay for Peptidylarginine deiminase activity (ABAP) kit. PADI3α and PADI3β *n* = 4, PADI3α + β *n* = 3; N.D., not detected; data are represented as mean ± s.d.; **p < 0.01 using unpaired Student's *t*-test. Graph is representative of three independent experiments. (*g*) Triple transfection of PADI3α-HA, PADI3α-Flag and PADI3β-His into HEK293 cells followed by co-immunoprecipitation for PADI3α-HA (upper panel, left) and PADI3β-His (upper panel, right) 48 h after transfection. The presence of the PADI3α-Flag expression (lower panel) in both PADI3α-HA and PADI3β-His pulldowns indicate the formation of homodimers (PADI3α-HA:PADI3α-Flag) as well as heterodimers (PADI3β-His:Padi3α-Flag). (*h*) Western blot analysis of PADI3α-HA, PADI3β-His, PADI3β-HA, and co-expression of PADI3a-HA and PADI3β-His. HA is probed to visualize the protein abundance of each isoform alone and the effect of co-expression (top panel). To avoid cross-signal reactivity of HA and His, PADI3β-His is blotted on another membrane (lower panel). The same cell lysates were run on two parallel gels and membranes for selective HA-tag and His-tag blotting. GFP indicates successful and equal transfection, and histone H3 is used as an internal loading control for each individual membrane. (*i*) Three-dimensional models comparing the structure of PADI3α homodimers to PADI3α-PADI3β heterodimers (arrows, purple and green, respectively) [36,37].

We show that both *Padi3α* and *Padi3β* mRNA is found in hair follicle progenitor and lineage committed cells during development (figure 3). Visualizing *Padi3* promoter activity using the published *Padi3*-LacZ reporter [29] supported PADI3 enrichment in the inner differentiated layers of the hair follicle (hair shaft and IRS). By contrast, LacZ-reporter activity was reduced in the ORS, corresponding largely to the FACS isolated ITGα6-high hair follicle cell population (figures 3*d* and 4*a*). To address if *Padi3α* and *Padi3β* transcripts are co-expressed throughout the hair follicle lineage or spatially restricted to discrete cell states during lineage progression, we designed isoform-specific *in situ* hybridization probes and visualized *Padi3α* and *Padi3β* in developing hair follicles (figure 4*b*). We found that *Padi3α* and *Padi3β* transcripts were expressed by hair follicle progenitor cells as well as differentiated lineages (figure 4*c*). Whereas the *in situ* signal for *Padi3α* was more pronounced compared to *Padi3β* intensity and abundance*,* indicating that *Padi3α* is the predominant transcript expressed in developing hair follicles, both transcripts were found at low levels in proliferating progenitor cells only to increase in expression concomitant with lineage specification and differentiation (figure 4*c*). The spatial *Padi3α* and *Padi3β* distribution supports previous reports demonstrating PADI3 protein expression and citrullination in the hair follicle [29,41–44] and suggests that *Padi3α* and *Padi3β* are co-expressed in the hair follicle lineage.

Given the expression profile of PADI3α and PADI3β, we aimed to explore the function of PADI3β. *In vitro* overexpression of PADI3α and PADI3β allowed us to probe and compare PADI3α and PADI3β in parallel. Although PADI3 is normally found in the cytoplasm, removal of the C-terminal domain shifts the PADI3 distribution towards the cell nucleus [45], suggesting that specific PADI domains confer subcellular enrichment preference. To understand if the N-terminal substitution of PADI3β affects protein distribution, we transfected HEK293 cells with PADI3α-Flag, PADI3α-HA, PADI3β-HA and PADI3β-His. All backbones (except for Flag) were bicistronic, expressing PADI3 as well as GFP as a positive marker for transfection. Localization of tagged PADI3α, PADI3β and GFP 48 h after transfection revealed a predominantly cytoplasmic PADI3α (Flag or HA) and PADI3β (His or HA) distribution in GFP positive transduced cells (figure 4*d*). These results indicate that PADI3 cellular localization is unaffected by the PADI3β N-terminal substitution.

### (e) PADI3β is unable to mediate citrullination

Mutations in human PADI3 affect enzymatic activity [29]. We, therefore, asked if PADI3α or PADI3β were able to citrullinate proteins when overexpressed in HEK293 cells. Interestingly, 3 h of ionomycin and calcium treatment induced prominent citrullination, detected using western blot, in cells transfected with PADI3α, but not PADI3β. In addition, co-transfection of PADI3α and PADI3β reduced the amount of citrullinated proteins detected (figure 4*e*). Using an antibody-based assay to measure the enzymatic capacity of PADIs *in vitro* [46], we confirmed the efficient citrullination potential of PADI3α as well as the inability of PADI3β to mediate citrullination. Furthermore, co-expression of PADI3α and PADI3β reduced the overall enzymatic capacity (figure 4*f*). Taken together, these data show that not only is PADI3β unable to induce citrullination, but PADI3β negatively affects the enzymatic output of PADI3α.

### (f) PADI3α and PADI3β binding affects protein stability

PADI3 homodimerizes to achieve full enzymatic activity [47]. Since co-expression of PADI3β and PADI3α reduced PADI3α-mediated citrullination, we speculated that PADI3β could bind to PADI3α. Co-transfection of PADI3α-Flag, PADI3α-HA and PADI3β-His to HEK293 cells allowed us to probe for PADI3α-to-PADI3α (Flag-to-HA) as well as PADI3α-to-PADI3β (Flag-to-His) binding. As expected, samples immunoprecipitated for PADI3α*-HA* were positive for PADI3α-Flag demonstrating PADI3α-to-PADI3α binding. Notably, immunoprecipitation for PADI3β-His also resulted in the pulldown of PADI3α-Flag, indicating a PADI3α-to-PADI3β interaction (figure 4*g*). These data suggest that PADI3β and PADI3α are able to interact when co-expressed.

While immunoprecipitation is able to enrich for His-tagged PADI3β, we fail to see prominent His-expression in the input sample, suggesting that the overall PADI3β protein levels are low, in line with the reduced protein stability resulting from PADI3 mutations [29] or truncation of PADI2 (figure 2*d*). To this end, we transfected HEK293 cells with PADI3α-HA, PADI3β-HA and PADI3β-His individually, as well as PADI3α-HA and PADI3β-His together. GFP was used as a proxy for transfection efficiency. Probing for the HA-tag we found that the amount of PADI3β protein was notably lower compared to PADI3α and that PADI3α levels decrease when co-expressed with PADI3β (figure 4*h*). Comparing the His-tag intensity for PADI3β in single or co-transfected cells, revealed that PADI3β levels also decreased during co-expression with PADI3α (figure 4*h*) suggesting that although PADI3β is able to interact with PADI3α, the PADI3β isoform is relatively unstable. Taken together, our data suggest that PADI3α can complex with PADI3β and that the presence of PADI3β reduces the stability of PADI3α (figure 4*i*).

## 3. Discussion

The ability of protein isoforms to modulate stem cell lineage progression and impact disease is well established. PADI enzyme isoforms are predicted but have never been verified or characterized *in vivo*. Here, we identify expression of *Padi2α* and *Padi2β* in the oligodendrocyte lineage and find that PADI2α and PADI2β differ in protein stability and ability to affect oligodendrocyte differentiation [12]. In addition, we probe the expression and localization of *Padi3α* and *Padi3β* isoforms and find that they are co-expressed in hair follicle progenitor cells committing to lineage differentiation. We demonstrate that although both PADI3α and PADI3β localize to the cytoplasm, only PADI3α enzymatic activity is detected. Furthermore, the PADI3β isoform is unstable and able to reduce the stability of PADI3α when heterodimerized, hereby likely contributing to the diminished enzymatic activity seen in HEK293 cells co-expressing both isoforms.

PADI enzymes are well conserved between orthologues as well as species [28,48,49] rendering it possible to extrapolate protein domain function between PADI members. The N-terminal domain, truncated or altered in both PADI3β and PADI2β, is shown to be important for protein–protein interactions [27,28]. PADI2α regulates expression and chromatin accessibility of genes associated with oligodendrocyte differentiation [12]. Here, we demonstrate that silencing of PADI2β promotes oligodendrocyte differentiation (figure 2), suggesting that PADI2β opposes the action of PADI2α. While the PADI2β mechanism of action is unknown, it is possible that the N-terminally truncated PADI2β lacks amino acid residues required for interaction with DNA-binding cofactors normally directing PADI2α to oligodendrocyte lineage-specific promoters. Alternatively, PADI2β could attain new functionality by acquiring binding affinity to new sets of effectors and subsequently access to additional target genes, counteracting the action of PADI2α. Considering the low protein stability of PADI2β, we speculate that the impact of PADI2β is transient and acting to fine-tune lineage commitment.

The N-terminal domain is also involved in PADI dimerization. While we did not directly investigate the dimerizing ability of either PADI2β or PADI3β, our work suggests that PADI3β is able to bind to PADI3α (figure 4), indicating that in PADI3β protein binding is unaffected by the N-terminal changes. Interestingly, the PADI3β isoform is not able to mediate citrullination in our hands (figure 4*e*,*f*), despite maintaining an intact C-terminal domain normally essential for catalytic activity. We cannot exclude that the low PADI3β protein stability, rather than intrinsic lack of enzymatic activity, explains the lack of citrullination. In addition, we find that co-expression of PADI3β and PADI3α reduces the overall enzymatic activity, and stability, of PADI3α. These data suggest that PADI3β modulates the effect of PADI3α, potentially through establishing PADI3α–PADI3β heterodimers.

Our data indicate that PADI2β and PADI3β isoforms are unstable, and both proteins fail to accumulate efficiently when overexpressed (figures 2*d* and 4*h*). It is possible that PADI2β and PADI3β stability is enhanced when bound to other proteins, be it PADIs or other interactors. Expression of unstable PADI isoforms could thus facilitate transient and dynamic regulation of citrullination, where the functional PADI outcome would be dependent of the cellular context. Considering the transcriptional and chromatin rewiring associated with progenitor lineage differentiation, cell state-specific citrullination profiles are likely to contribute to lineage progression. Using mRNA depletion strategies [50], we are now in a position where specific PADI isoforms can be silenced *in vivo*, paving the road for functional studies elucidating tissue phenotype as well as isoform-specific citrullinomes and substrate specificities.

The work presented herein unveils the previously uninvestigated strategies PADIs could undertake to regulate tissue formation while unearthing new questions. Given the finding that the alternative isoforms of PADI2β and PADI3β can modulate the function of the conventional, prevailing PADI2α/PADI3α, it will be interesting to determine expression of additional protein-coding PADI isoforms in both mice and humans. PADIs are reported to be deregulated in disease [18–20,41,51], but whether certain PADI isoforms are enriched in, or associated with, human pathology remains to be seen. Here, we identify and functionally describe previously uncharacterized *Padi2* and *Padi3* isoforms *in vivo*, conceptually advancing our understanding of how citrullination can impact tissue formation.

## 4. Material and methods

### (a) Mouse husbandry

The mouse lines used in this study were *Pdgfra:*H2B-GFP [52], *Pdgfr*a:Cre (The Jackson Laboratory mouse strain 013148) crossed to the RCE:loxP mice harbouring the R26R CAG-boosted EGFP (The Jackson Laboratory mouse strain 032037-JAX) [53] *Krt14*:H2B-GFP [39] and Padi3^tm1a^ :LacZ reporter [29]. Females with a hemizygous Cre allele were mated with males lacking the Cre allele, while the reporter allele was kept in hemizygosity or homozygosity in both females and males. All animals were free from mouse viral pathogens, ectoparasites and endoparasites and mouse bacteria pathogens. Mice were kept with the following light/dark cycle: dawn 06.00–07.00, daylight 07.00–18.00, dusk 18.00–19.00, night 19.00–06.00 and housing to a maximum number of five per cage in individually ventilated cages (IVC sealsafe GM500, tecniplast). General housing parameters such as relative humidity, temperature and ventilation follow the European convention for the protection of vertebrate animals used for experimental and other scientific purposes treaty ETS 123, Strasbourg 18.03.1996/01.01.1991. Briefly, consistent relative air humidity of 55 ± 10%, 22°C and the air quality is controlled with the use of stand-alone air-handling units supplemented with HEPA filtrated air. Monitoring of husbandry parameters is done using ScanClime (Scanbur) units. Cages contained hardwood bedding (TAPVEI, Estonia), nesting material, shredded paper, gnawing sticks and a card box shelter (Scanbur). The mice received a regular chow diet (either R70 diet or R34, Lantmännen Lantbruk, Sweden). Water was provided by using a water bottle, which was changed weekly. Cages were changed every other week. All cage changes were done in a laminar air-flow cabinet. Facility personnel wore dedicated scrubs, socks and shoes. Respiratory masks were used when working outside of the laminar air-flow cabinet. Animals were sacrificed at postnatal, juvenile, and adult stages and both sexes were included in the study. All experimental procedures on animals were performed following the European directive 2010/63/EU, local Swedish directive L150/SJVFS/2019:9, Saknr L150 and Karolinska Institutet complementary guidelines for procurement and use of laboratory animals, Dnr. 1937/03-640. The procedures described were approved by the regional committee for ethical experiments on laboratory animals in Sweden (Stockholms Norra Djurförsöksetiska nämnd), Lic. no. 131/15, 144/16, 1995/2019 and 14051-2019.

### (b) FACS isolation of hair follicle populations

Back skins from *Krt14*:H2B-GFP mice at P2 were dissected and put in HBSS (Thermofisher Scientific (Gibco)) with 20% collagenase (in PBS (Sigma-Aldrich)) for 1 h at 37°C, after which the dermal/hair follicle fraction was scraped off and collected using centrifugation (300*g* for 10 min). The pellet was suspended in trypsin and incubated for 15 min at 37°C. Cell suspensions were filtered using both 70 and 40 µm filtres, washed twice in PBS and pelleted using centrifugation for 5 min at 300*g* [40]. Epithelial hair follicle cells were H2B-GFP positive. Interfollicular epidermal cells were excluded using SCA-1 PerCP-C5.5 and two hair follicle cell populations were isolated based on high/low CD49f (*α*6 integrin [*IT*Gα6])-PE expression. DAPI was used to exclude dead cells. Using a fluorescence-activated cell sorter (BD FACS AriaII), sorted cells were collected in Trizol LS (Thermofisher (Invitrogen)) reagent and stored at –80°C until RNA extraction/cDNA synthesis.

### (c) FACS isolation of oligodendrocyte precursors and oligodendrocytes

Brain tissue isolated from postnatal (P4), juvenile (P21) and adult (P60) *Pdgfrα*:Cre-RCE:loxP-GFP mice was dissociated to single cells with neural (for postnatal) and adult (for juvenile and adult) tissue dissociation kits (P; Miltenyi Biotec) without the red blood cell removal step. Cell suspensions were then filtered with a 30 mm filter (Partec). For OPC removal from OL lineage cells from juvenile and adult brains, CD140a (anti-mouse CD140 APC conjugated, BD Bioscience) labelling was performed. GFP+ cells from postnatal brains and GFP+/CD140a- cells from juvenile and adult brains were isolated with fluorescence-activated cell sorting (FACS) using a BD FACSAria III Cell Sorter B5/R3/V3 system. Sorted cells were collected in Eppendorf tubes, centrifuged at 400*g*, resuspended in Qiazol and stored at −80°C until RNA extraction/cDNA synthesis.

### (d) PADI3α and PADI3β cloning

The coding sequence of mouse PADI3α (Q9Z184) or PADI3β (E9QAM4) with C-terminus fused to Myc-3xFlag, HA or 6xHis-tag, was cloned into pUC19 backbone (pRP[Exp]-CMV>:IRES-eGFP, manufactured by VectorBuilder) via In-Fusion Snap Assembly (Takara).

### (e) Culture and transfection of HEK293 cells

HEK293 cells were grown in DMEM (Fisher Scientific, 41966029) supplemented with 10% FBS (Sigma-Aldrich) and 1% penicillin/streptomycin (Thermofisher Scientific (Gibco)). For western blot, cells were transfected in six-well plates at approximately 80% confluency with 500 ng of either Myc-3xFlag-PADI3α, HA-PADI3α-IRES-eGFP, HA-PADI3β-IRES-eGFP or 6xHis-PADI3β-IRES-eGFP plasmid DNA using Lipofectamine LTX with PLUS reagent (Thermofisher Scientific, 15338100) according to manufacturer's instructions. For immunofluorescence analysis, HEK293 cells were transfected using Effectene Transfection Reagent (Qiagen, 301425) according to manufacturer's instructions for the Fast-forward protocol for transient transfection of 293 cells in 96-well plates.

### (f) Culture of primary OPCs

Brains from postnatal day 4 (P4) *Pdgfrα*:H2B-GFP hemizygous pups were isolated and dissociated with the neural tissue dissociation kit (P; Miltenyi Biotech 130-092-628). GFP+ OPCs were isolated with FACS and collected in proliferation medium (DMEM/F12 glutamax (Gibco-Life Technologies 10565018) containing NeuroBrew 21 (B27; Miltenyi Biotech 130-093-566); penicillin–streptomycin (Life Technologies 15140122); N2 supplement (Life Technologies 17502048); PDGF-αα (R&D Systems 520-BB-050; 10 ng ml^−1^); bFGF (PeproTech 100-18B; 20 ng ml^−1^)). Plates were coated with poly-l-lysine (Sigma-Aldrich, P4707) overnight and fibronectin (Sigma-Aldrich F1141; 1 : 1000) for 1 h at 37°C. Cells were differentiated with differentiation medium (DMEM/F12 glutamax containing NeuroBrew 21 (B27); pencillin–streptomycin; N2 supplement; T3 (Sigma-Aldrich T6397; 40 ng ml^−1^)) for 2.5 days.

### (g) Culture of Oli-neu cells

Embryonic mouse oligodendrocyte precursor cells (Oli-neu) were grown in DMEM (Gibco-Life Technologies 41965062) containing N2 supplement (Life Technologies), penicillin–streptomycin–glutamine (Life Technologies 10378016), T3 (Sigma-Aldrich; 340 ng ml^−1^), T4 (Sigma-Aldrich 89430; 400 ng ml^−1^), bFGF (PeproTech; 10 ng ml^−1^) and PDGF-ββ (R&D Systems; 1 ng ml^−1^) on plates coated with poly-l-lysine (Sigma-Aldrich; 0.01%). Cells were differentiated with differentiation medium (DMEM containing N2 supplement; penicillin–streptomycin–glutamine; T3 (Sigma-Aldrich; 340 ng ml^−1^); T4 (Sigma-Aldrich; 400 ng ml^−1^); ErbB inhibitor (Santa Cruz sc-204170; 1 µM)) for 1 day.

### (h) PADI2β overexpression and silencing

*PADI2β* and control overexpression cell lines were generated by insertion of the following vectors with piggyBac transposon system: pB-CAG-Ctr, pB-CAG-PADI2β for the control and overexpression of *PADI2β*, respectively; the gateway system was used to clone these vectors in the final piggyback plasmids (Gateway LR Clonase II Enzyme mix, Invitrogen/ThermosFisher Scientific, 11791020). pB-CAG-Ctr, pB-CAG-*PADI2β* were transfected together with piggyBac transposase (pBase) expression vector by lipofection according to the manufacturer's instructions (Lipofectamine 2000, Invitrogen 11668019). All cell lines express the hygromycin resistance gene in the DNA integrated vectors and were selected and expanded in media containing 200 mg ml^−1^ 1 of hygromycin (Hygromycin B gold ant-hg-1, InvivoGen).

For silencing experiments, cells were plated on a 12-well plate 1 day prior to lipofection. One microgram of siRNA was diluted in OPTIMEM and mixed with Lipofectamine L2000 (Invitrogen), following manufacturer's instructions. For primary OPCs, after 4 h of adding the lipid/DNA complexes to the cells, media was changed to either proliferation or differentiation media and cells were collected 2.5 days after. For Oli-neu experiments, after 4 h of adding the lipid/DNA complexes to the cells, media was changed to proliferation for 1 day and cells were either collected at this point (proliferation condition) or changed to differentiation media one more day (differentiation condition). siRNA was purchased from Dharmacon GE Healthcare, using the siDESIGN centre, with the following sequences:

– mouse *Padi2β*; sense: 5′ GGGAGAAACUUGAUAACUUUU 3′; antisense: 5′ AAGUUAUCAAGUUUCUCCCUU 3′
– mouse *Padi2a;* sense: 5′ CGUACGUGAUGGAGAGGCAUU 3′; antisense: 5′ UGCCUCUCCAUCACGUACGUU 3′
– mouse siCtrl (for *Padi2a* and *Padi2β*); ON-TARGETplus Non-targeting siRNA #1 (D-001810-01-20)
– mouse *Padi2 3′UTR*; sense: 5′ CCAAUAAGCAUACGCUCAA 3′; siGENOME (D-062139-13-0005)
– mouse siCtrl (for *Padi2 3′UTR*); siGENOME non-targeting Control siRNA #1 (D-001210-01-05).

### (i) PCR walking for Padi2β

For PCR walking, primers were designed from the predicted *Padi2β* cDNA 5′ end. The following primers were used:

Padi2β_ex1_Forward: 5′ GAAAGCAGCCCCAAATAGAAGAT 3′;

Padi2_ex14_Reverse: 5′GAGGCTCTCATTGGACAGGA 3′;

Padi2_ex15_Reverse: 5′TCTTAAGGATGTCGCGGTTC 3′;

Padi2_ex16_Reverse: 5′AAGTTGGTACAACCCAGCCA 3′.

PfuUltra II fusion High-Fidelity DNA Polymerase (Agilent) was used to amplify cDNA fragments of *Padi2β* from an Olineu cDNA pool. The pairs of primers used were Padi2β_ex1_Forward with Padi2_ex14_Reverse,

Padi2β_ex1_Forward with Padi2_ex15_Reverse

Padi2β_ex1_Forward with Padi2_ex16_Reverse.

The PCR products were run in a 2% agarose gel and the expected size bands were cut from the gel under a UV light. The PCR products were extracted and purified using a QIAquick PCR gel purification kit (Qiagen 28794) and sent for sequencing.

### (j) RNA extraction, cDNA synthesis and quantitative real-time PCR

RNA was extracted using the RNAeasy kit (Qiagen), miRNeasy mini kit (Qiagen) (hair follicle (HF) cells) or miRNeasy micro kit (Qiagen) (primary OPCs) according to manufacturer's protocols. Total HF RNA (100 ng) was used for cDNA synthesis using SuperScript VILO (Thermo Fisher, 11754050); 350 ng RNA (primary OPCs) was used to synthesized cDNA using the High-Capacity cDNA Reverse Transcription Kit (Applied Biosystems) including RNase inhibitor (Applied Biosystems). RT-qPCR for HF populations was run with selected primer pairs and SYBR Green on a ViiA 7 device or a 7500 fast system (both Applied Biosystems). *Hprt* was used as an internal control. cDNA from primary OPC samples were analysed on a StepOnePlus System (Applied Biosystems) in duplicate and with reverse transcriptase negative reactions to control for genomic DNA. *Tbp* and *Gapdh* or *Ubc* were run as housekeeping genes and relative standard curves were generated for each gene to determine relative expression.

### (k) immunofluorescence OPCs

Differentiated OPCs were fixed in 4% formaldehyde for 10 min, washed in PBS and incubated overnight at 4°C with the primary antibodies anti-MOG (Millipore, MAB5680, 1 : 200) and anti-PDGFRα (R&D Systems, AF1062, 1 : 200) in PBS/0.5% Triton/10% normal donkey serum (Sigma-Aldrich). Cells were washed with PBS and then incubated for 2 h at room temperature with Alexa Fluor-conjugated antibodies (Invitrogen, Alexa Fluor anti-goat 488, 1 : 1000 and Alexa Fluor anti-mouse 555, 1 : 1000). Cells were then mounted with mounting medium containing DAPI (Vector, H-1200).

### (l) Immunofluorescence HEK293s

Glass chamber slides were coated with Laminin-521 (Biolamina, LN521-02) overnight at 4°C prior to plating. Cells were fixed using 4% formaldehyde (Sigma-Aldrich) and then blocked with goat serum (2.5%) (Jackson ImmunoResearch) and BSA (1%) (Sigma-Aldrich) and permeabilized with 0.3% Triton X-100 (Sigma-Aldrich). Samples were incubated overnight with primary antibodies at 4°C, followed by incubation with Alexa Fluor-conjugated secondary antibodies (488 anti-chicken, Cy3 anti-mouse/rat/rabbit, Jackson ImmunoResarch) for 1 h at room temperature. DAPI was used for nuclear stain, and slides were mounted using ProLong Gold antifade (Thermofisher Scientific). Antibodies used for immunofluorescence staining were: anti-HA (Sigma-Aldrich, 11867423001, 1 : 1000), anti-Flag (Sigma-Aldrich, M2-F1804, 1 : 1000), anti-His (Abcam, ab9108, 1 : 1000) and anti-GFP (Aves, GFP-1020, 1 : 1000).

### (m) Western blot for PADI2 in Oli-neu cells

Cells were collected in 2× Laemmli buffer (120 mM Tris–HCl, pH 6.8; 4% SDS; 20% glycerol) and sonicated for 5 min at high power with 30 s on/off cycles to shear genomic DNA at 4°C. Protein concentrations were determined on nanodrop and concentrations were equalized with 2× Laemmli buffer. Bromophenol blue (0.01%) and β-mercaptoethanol (10%) were added to the samples and the samples were boiled at 95°C for 5 min to denature the protein. Equal volumes were loaded in an SDS-PAGE for protein separation and transferred to a PVDF membrane (GE Healthcare) activated in methanol. The membranes were blocked in blocking solution (TBS; 0.1% Tween 20; 5% milk) for 1 h at room temperature and incubated overnight with primary antibody (diluted in blocking solution) at 4°C, washed three times 10 min in TBS-T (TBS; 0.1% Tween 20) and incubated with a horseradish peroxidase (HRP)-conjugated secondary antibody (diluted in blocking solution) for 2 h at room temperature. Proteins were exposed with either enhanced chemiluminescence solution (GE Healthcare) or Supersignal west femto (Thermofisher Scientific) at a ChemiDoc XRS imaging system (Bio-Rad). Primary antibodies were used against PADI2 (rabbit polyclonal; 12110-1-AP; ProteinTech; 1 : 300) and histone H3 (mouse monoclonal; ab10799; Abcam; 1 : 1000). Secondary antibodies were used at a dilution of 1 : 5000, anti-rabbit (A6667; Sigma-Aldrich), anti-mouse (A4416; Sigma-Aldrich). The proteasomal inhibitor MG132 (10 µM) was used to enrich for PADI2β.

### (n) Western blot and immunoprecipitation for PADI3 isoforms in HEK293 cells

PADI3α and PADI3β were overexpressed by transfecting Myc-Flag/HA/6xHis-tagged constructs into HEK293 cells. Cells were harvested at 48 h post transfection in 1 ml ice-cold PBS, centrifuged at 900*g* at 4°C and pellets were resuspended in RIPA lysis buffer containing protease inhibitors and lysed for 1 h on ice. Debris was removed by centrifugation at 13 000*g* for 15 min at 4°C, and cleared lysates were prepared with 6x Laemmli SDS buffer (VWR) and boiled at 95°C for 5 min. Samples were run on an SDS/PAGE gel (Bio-Rad) and transferred onto a polyvinylidene difluoride (PVDF) membrane (Merck Millipore). Membranes were blocked with 5% milk in TBS containing 0.1% Tween-20 for 1 h at room temperature, incubated with primary antibody at 4°C overnight, followed by 1 h incubation with HRP-conjugated secondary antibody at room temperature. Blots were developed using SuperSignal West Femto Maximum Sensitivity Substrate (Thermofisher Scientific) and imaged with ChemiDoc (Bio-Rad).

The Myc-3xFlag/6xHis/HA-tagged protein was precipitated using anti-Flag magnetic agarose beads (Fisher Scientific), anti-HA-tag magnetic beads (Fisher Scientific), or Dynabeads His-tag Isolation and pulldown (Fisher Scientific, 10470515), respectively. Efficiency of precipitations was confirmed using western blot. In brief, 20 µl of 6x Laemmli buffer (VWR) was added to 100 µl of immunoprecipitated tagged proteins, followed by heating at 95°C for 5 min. The samples were then separated using 4–20% precast protein gels (Bio-Rad) and subsequently used for immunoblotting. The blot was developed using SuperSignal West Femto Maximum Sensitivity Substrate (Thermofisher Scientific) and imaged with ChemiDoc (Bio-Rad). Antibodies used for blotting include anti-HA (Sigma-Aldrich, 11867423001, 1 : 1000), anti-Flag (Sigma-Aldrich, M2-F1804, 1 : 1000), anti-His (Abcam, ab9108, 1 : 1000), anti-GFP (Aves, GFP-1020), 1 : 1000, anti-GAPDH (0411, SantaCruz, 1 : 1000) and anti-histone H3 (1791, Abcam, 1 : 1000). Secondary antibodies used were HRP-conjugated goat anti-rabbit, goat anti-chicken, donkey anti-rat and donkey anti-mouse at 1 : 10 000 (Jackson ImmunoResearch).

### (o) Detection of modified citrullinated residues using western blot

Forty-eight hours post-transfection, HEK293 cells were treated with 5 mM CaCl_2_ (Sigma-Aldrich, in dH_2_O) and 5 µM ionomycin (Thermofisher (Invitrogen), in DMSO), or DMSO for control, for 3 h after which the cells were collected and processed for western blot as described above. The Anti-Modified Citrulline Detection kit (17-347B) was used to assess the presence of citrullinated proteins according to the manufacturer's instructions. In brief, the PVDF membrane was incubated with a 1 : 1 mixture of Reagent A (0.025% FeCl_3_; 25% H_2_SO_4_; 17% H_3_PO_4_) and Reagent B (0.5% 2,3-butanedione monoxime; 0.25% antipyrine; 0.5 M acetic acid) overnight at 37°C without agitation in an airtight, lightproof container. The membrane was then rinsed six times with water and blocked with 5% milk in TBS containing 0.1%Tween-20 for 1 h at room temperature, incubated overnight with the primary Anti-Modified Citrulline Antibody, diluted in blocking solution (TBS; 0.1% Tween-20; 5% milk), at 4°C, followed by 1 h incubation with the anti-human IgG HRP-conjugated secondary antibody (also in blocking solution) at room temperature. Blots were developed using SuperSignal-Aldrich West Femto Maximum Sensitivity Substrate (Thermofisher Scientific, 34095) and imaged with ChemiDoc (Bio-Rad).

### (p) Antibody based assay for PADI3 isoform activity

HEK293 cell lysates were isolated 48 h after transfection as described for western blot. The Antibody Based Assay for PAD activity (ABAP) kit (Modiquest Research, MQ17.101-96) was used to assess the activity of PADI3α and PADI3β, either alone or in conjunction, and performed according to the manufacturer's instructions. In brief, a total of 10 µl of lysate was diluted in 100 µl of deimination buffer (40 mM Tris–HCl, pH 7.5; 5 mM CaCl_2_; 1 mM DTT) per well. Two independent samples for each isoform were used and loaded in duplicates. HRP-conjugated secondary antibody was developed with a TMB substrate solution (Thermfisher Scientific, N301; 0.1 M sodium acetate, pH 5.2; 0.01% hydrogen peroxide) and the reaction was stopped with Stop Solution (Thermofisher Scientific, N600; sulfuric acid (2 M H_2_SO_4_)). The absorbance at 450 nm was captured with a VersaMax microplate reader (Molecular Devices). Human PADI4 enzyme was used as control for enzymatic activity and was diluted in deimination buffer with concentrations between 0.002 mU and 2.0 mU (minimum to maximum deimination) to create a standard curve to correlate activity to the optical density measured at 450 nm.

### (q) Basescope *in situ* hybridization

RNA *in situ* hybridization to probe *Padi3α* and *Padi3β* was conducted using a BaseScope Detection Reagent Kit – RED from Advanced Cell Diagnostics (ACD), according to the manufacturer's instruction for fresh frozen samples. In brief, back skin of P2 pups were collected within 5 min of animal sacrifice, frozen in OCT (Tissue-Tek) and stored at –80°C until sectioning. Within 24 h, samples were sectioned at –20°C in a cryostat and mounted on room temperature SuperFrost Plus glass slides and left to dry in the cryostat for 1 h. Slides were immersed in ice-cold 4% formaldehyde (Sigma-Aldrich) and incubated for 40 min at 4°C, and then dehydrated using a series of increasing concentrations of ethanol at room temperature, 5 min each; 50%, 70%, and three times with 100% ethanol. All at room temperature, the slides were dried for 5 min followed by incubation with hydrogen peroxide for 10 min, and then treated with protease for 30 min, followed by incubation with Padi3α or Padi3β probes for 2 h at 40°C. After amplification with AMP 0-6, the signal was detected using BaseScope RED working solution (a 1 : 60 mix of BaseScope Fast RED-B to Fast RED-A), and counterstained with haematoxylin (Sigma-Aldrich) and 0.02% ammonium hydroxide (ammonia hydroxide solution, Sigma-Aldrich, in dH_2_O). The slides were then dried at 60°C for 15 min before being mounted with VectaMount media and air-dried for approximately 5 min.

### (r) Statistical analyses

GraphPad Prism have been used for statistical analyses. Error bars show the s.d. as specified in the figure legends.

## Data Availability

The data are provided in the electronic supplementary material [54].
